# Author Correction: Tracking moving objects through scattering media via speckle correlations

**DOI:** 10.1038/s41467-022-33949-8

**Published:** 2022-10-17

**Authors:** Y. Jauregui-Sánchez, H. Penketh, J. Bertolotti

**Affiliations:** Physics and Astronomy Department, University of Exeter, Stocker Road, Exeter, EX4 4QL UK

**Keywords:** Imaging and sensing, Optical physics

**Correction to**: *Nature Communications* 10.1038/s41467-022-33470-y, published online 01 October 2022

The original HTML version of this Article was updated shortly after publication because the previous HTML version linked to the LaTeX source file of the Supplementary Information. This has been replaced by the PDF version of the file.

## Supplementary information

Updated Supplementary Information

