# Supplementary material for: Tracking moving objects through scattering media via speckle correlations
Source: Nat Commun. 2022 Oct 1;13:5779. doi: 10.1038/s41467-022-33470-y (PMC9526741; doi:10.1038/s41467-022-33470-y)
Supplement: Supplementary file 1 — Supplementary Information [file 41467_2022_33470_MOESM1_ESM.pdf]

Supplementary Information: Tracking moving objects through scattering media via speckle correlations

Y. Jauregui-Sánchez, H. Penketh, and J. Bertolotti
Physics and Astronomy Department, University of Exeter,
Stocker Road, Exeter EX4 4QL, UK

S1 The autocorrelation of the intensity

A point source at $\mathbf{x}_\mathrm{o}$ will produce an intensity at $\mathbf{x}_\mathrm{d}$ given by the speckle pattern $S(\mathbf{x}_\mathrm{o}, \mathbf{x}_\mathrm{d})$. Notice that here S behaves effectively as the Green's function for the scattering process [1].

$$I(\mathbf{x}_\mathrm{d}) = \int O(\mathbf{x}_\mathrm{o})\, S(\mathbf{x}_\mathrm{o}, \mathbf{x}_\mathrm{d}) \mathrm{d}^2\mathbf{x}_\mathrm{o}. \tag{S1}$$

If we take the autocorrelation of the measured intensity we obtain

$$\begin{aligned}
[I \star I](\boldsymbol{\Delta x}_\mathrm{d}) &= \int I(\mathbf{x}_\mathrm{d})\, I(\mathbf{x}_\mathrm{d} + \boldsymbol{\Delta x}_\mathrm{d}) \mathrm{d}^2\mathbf{x}_\mathrm{d} = \\
&= \int \left[\left(\int O(\mathbf{x}_\mathrm{o})\, S(\mathbf{x}_\mathrm{o}, \mathbf{x}_\mathrm{d}) \mathrm{d}^2\mathbf{x}_\mathrm{o} \right) \left(\int O(\mathbf{y}_\mathrm{o})\, S(\mathbf{y}_\mathrm{o}, \mathbf{x}_\mathrm{d} + \boldsymbol{\Delta x}_\mathrm{d}) \mathrm{d}^2\mathbf{y}_\mathrm{o} \right) \right] \mathrm{d}^2\mathbf{x}_\mathrm{d} = \\
&= \iint O(\mathbf{x}_\mathrm{o}) O(\mathbf{y}_\mathrm{o}) \left(\int S(\mathbf{x}_\mathrm{o}, \mathbf{x}_\mathrm{d})\, S(\mathbf{y}_\mathrm{o}, \mathbf{x}_\mathrm{d} + \boldsymbol{\Delta x}_\mathrm{d})\, \mathrm{d}^2\mathbf{x}_\mathrm{d} \right) \mathrm{d}^2\mathbf{x}_\mathrm{o} \mathrm{d}^2\mathbf{y}_\mathrm{o} = \\
&= \int O(\mathbf{x}_\mathrm{o})\, O(\mathbf{y}_\mathrm{o}) \Big([S \star S]\, (\mathbf{x}_\mathrm{o}, \mathbf{y}_\mathrm{o}, \boldsymbol{\Delta x}_\mathrm{d}) \Big) \mathrm{d}^2\mathbf{x}_\mathrm{o} \mathrm{d}^2\mathbf{y}_\mathrm{o}.
\end{aligned} \tag{S2}$$

We define $\delta S = S - \overline{S}$ with $\overline{S}$ the average intensity of the speckle pattern: $\overline{S} = \frac{\int S(\mathbf{x}_\mathrm{o}, \mathbf{x}_\mathrm{d})\, \mathrm{d}^2\mathbf{x}_\mathrm{d}}{A}$, where $A = \int \mathrm{d}^2\mathbf{x}_\mathrm{d}$ is the area covered by the speckle pattern. We assume that the speckle has the same statistical properties for

all $\mathbf{x}_\mathrm{o}$, and thus $\overline{S}$ does not depend on it. To go further, notice that the autocorrelation of S and the autocorrelation of δS differ just by a constant:

$$\begin{aligned}
&\delta S \star \delta S = \left(S - \overline{S}\right) \star \left(S - \overline{S}\right) = S \star S + \overline{S} \star \overline{S} - 2S \star \overline{S} = \\
&= \int S(\mathbf{x}_\mathrm{o}, \mathbf{x}_\mathrm{d})\, S(\mathbf{y}_\mathrm{o}, \mathbf{x}_\mathrm{d} + \boldsymbol{\Delta x}_\mathrm{d})\, \mathrm{d}^2\mathbf{x}_\mathrm{d} + \int \overline{S}^2\, \mathrm{d}^2\mathbf{x}_\mathrm{d} - 2\int S(\mathbf{x}_\mathrm{o}, \mathbf{x}_\mathrm{d})\, \overline{S}\, \mathrm{d}^2\mathbf{x}_\mathrm{d} = \\
&= S \star S - \overline{S}^2 A \quad \Rightarrow S \star S = \delta S \star \delta S + \overline{S}^2 A.
\end{aligned} \tag{S3}$$

Substituting equation S3 into equation S2 we obtain

$$\begin{aligned}
&[I \star I](\boldsymbol{\Delta x}_\mathrm{d}) = \int O(\mathbf{x}_\mathrm{o})\, O(\mathbf{y}_\mathrm{o}) \Big([\delta S \star \delta S]\, (\mathbf{x}_\mathrm{o}, \mathbf{y}_\mathrm{o}, \boldsymbol{\Delta x}_\mathrm{d}) \Big) \mathrm{d}^2\mathbf{x}_\mathrm{o} \mathrm{d}^2\mathbf{y}_\mathrm{o} + \\
&+ \overline{S}^2 A \int O(\mathbf{x}_\mathrm{o})\, O(\mathbf{y}_\mathrm{o}) \mathrm{d}^2\mathbf{x}_\mathrm{o} \mathrm{d}^2\mathbf{y}_\mathrm{o},
\end{aligned} \tag{S4}$$

which, performing an ensemble average over the realization of disorder $\langle . \rangle$, we can rewrite as

$$\langle I \star I \rangle\, (\boldsymbol{\Delta x}_\mathrm{d}) = \overline{S}^2 \int O(\mathbf{x}_\mathrm{o})\, O(\mathbf{y}_\mathrm{o}) \mathcal{C}(\mathbf{x}_\mathrm{o}, \mathbf{y}_\mathrm{o}, \boldsymbol{\Delta x}_\mathrm{d}) \mathrm{d}^2\mathbf{x}_\mathrm{o} \mathrm{d}^2\mathbf{y}_\mathrm{o} + \overline{S}^2 A\, \|O\|^2, \tag{S5}$$

where $\mathcal{C}$ is the correlations function $\mathcal{C} = \frac{\langle \delta S \star \delta S \rangle}{\overline{S}^2}$ [2], and

$$\|O\|^2 = \int O(\mathbf{x}_\mathrm{o})\, O(\mathbf{y}_\mathrm{o}) \mathrm{d}^2\mathbf{x}_\mathrm{o} \mathrm{d}^2\mathbf{y}_\mathrm{o}.$$

Finally, performing the change of variable $\mathbf{y}_\mathrm{o} = \boldsymbol{\Delta x}_\mathrm{o} + \mathbf{x}_\mathrm{o}$, we obtain the compact formula

$$\langle I \star I \rangle\, (\boldsymbol{\Delta x}_\mathrm{d}) = \overline{S}^2\, [O \star O] \otimes \mathcal{C} + \overline{S}^2 A \|O\|^2. \tag{S6}$$

Equation S6 shows that by measuring the speckle-like intensity I, performing an autocorrelation, and then averaging (either over many different scattering layers, or over a large speckle field), one obtains something linearly proportional to the autocorrelation of the unknown object O, convolved with the correlation function $\mathcal{C}$, which effectively behaves like a point spread function and determine the achievable resolution [3, 4]. The second term ($\overline{S}^2 A \|O\|^2$) represents a flat background that in principle can be subtracted, but one

has to be careful as mistakes in subtracting it can easily lead to artefacts in the reconstruction. Notice that, as this term is approximately constant with time, when we calculate $I(t_n) \star I(t_{n-1}) - I(t_n) \star I(t_n)$ it will automatically cancel.

S1.1 The ensemble average

Equation S6 relates the ensemble average of the autocorrelation of the measured intensity to the autocorrelation of the unknown object, but in the experiment, no ensemble average is performed [1,3]. As a consequence, the speckle autocorrelation $S \star S$ (and thus $\mathcal{C}$) did not yet fully converge to its ensemble average, and still has speckle-like fluctuations. It is important to notice that what counts for the average is how many different speckle spots one is averaging when making the autocorrelation, and if one measures a wide enough angular range, $\delta S \star$ is not too dissimilar from $\langle \delta S \star \delta S \rangle$.

S2 Experimental setup

The experiments were carried out on the setup shown in Supplementary Fig. S1. The moving scene was generated on a digital micromirror device (DMD, Texas Instruments DLP9000), illuminated uniformly with a Red He-Ne laser beam (HNLS008R, Thorlabs). The laser beam was expanded 10X by a Galilean telescope and passed through a rapidly rotating diffuser (RD) to reduce significantly the spatial coherence of the light source. The Galilean telescope was formed by a biconcave lens (L1, $f_1 = -25$mm) and a plano-convex lens (L2, $f_2 = 250$mm). To filter out the unwanted diffracted orders produced by the periodic arrangement of micromirrors in the DMD, a circular diaphragm was used. To mimic moving objects, a sequence of binary masks was coded on the DMD in a chip area of 2560×1600 micromirrors. The reflected light from the DMD was passed through a scattering medium

and recorded by a digital camera (Allied Vision Manta G-125B, Edmund Optics). The resolution of the image sensor was 1292×964 pixels with a pitch of 3.75 μm. The distances between the DMD, the scattering medium (L_o), and the camera sensor plane (L_s) were 430 mm and 50 mm, respectively. In the first part of the experiment, the moving scene was composed of three geometric shapes: a circle with a diameter of 306 μm, a triangle of 306×306 μm, and a rectangle of 306×456 μm. The first two geometric shapes remained static, while the rectangle moved and rotated. The scattering medium was a 220-grit ground glass diffuser (DG10-220-MD, Thorlabs). In the second one, the moving scene was made of a large number of static dots and a single moving star. In this case, two layers of adhesive sellotape were used as a scattering medium to reduce the memory range.

The camera images were post-processed as follows. First, a mean value was subtracted from the measurements to reduce the constant background (see equation S6). Second, smooth intensity variations due to non-uniform illumination of the camera were removed by fitting a second-order polynomial to the image and subtracting it. After that, the 2D Fourier transform of the measured speckle taken at time t_0 was multiplied by the complex conjugate of the 2D Fourier transform of the measured speckle taken at time t_n. Finally, the cross-correlation was obtained by calculating the inverse Fourier transform of the previous product. As the autocorrelation of white noise is always a spike in the central pixel, and this spike contains no information on the image, we removed it. The camera images were recorded for an exposure time of 3 seconds and cropped into a square window of 512×512 pixels (see MATLAB code at https://doi.org/10.5281/zenodo.6124320).

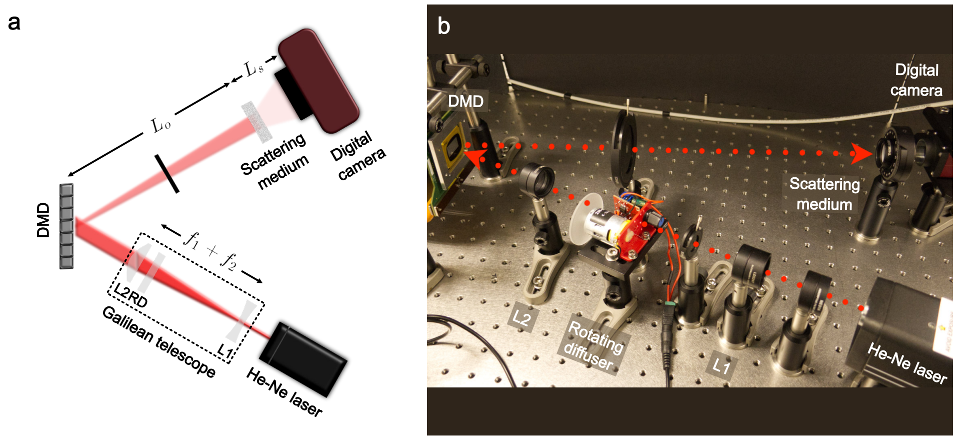

Figure S1: **a** Schematic representation of the experimental setup in **b** used to take the measurements.

S2.1 Signal to noise considerations

As white noise is delta-correlated, when performing an autocorrelation the camera noise transforms into a sharp spike in the centre of the image, which can be safely removed [3]. Furthermore, the cross-correlation between two white noise patterns is zero. So, as long as the camera noise is not too big with respect with the measured signal, it doesn't effect the tracking ability. A subtler, but more important limiting factor in how well we can track the movement of an object is due to the imperfect ensemble average of the speckle correlation $\mathcal{C}$, as discussed in section S1.1. This produces a low-amplitude speckle-like pattern on the bottom of each frame (as visible in Fig. 3c). Performing the difference in equation 7 helps averaging them out a bit, but the same difference means that the signal we are looking for is small, especially if the object did not move much between two frames. In practical terms, in order for the signal extracted from equation 7 to be visible over the speckle-like background, we need the camera to be sensitive enough to be able to detect the changes in the speckle intensity I when the object moves.

S3 Tracking of a reflective object

To test the tracking of real moving objects, instead of using a sequence of binary mask coded on the DMD, we made an object comprised of three small (~0.5 mm diameter) planar reflective hexagons, from a biodegradable glitter. Two of these sit upon a stationary black card substrate as shown in Supplementary Fig. S2a whilst one has a substrate of reduced area that sits atop the other and is connected to a linear translation stage. The stage allows the third hexagon to be moved horizontally from the others whilst remaining in a parallel plane to them. As shown in Supplementary Figures S2b and S2c, the moving reflecting object can be tracked as well as the images projected on the DMD (see Supplementary Video 6). Notice that this approach has infinite depth of field, so the fact that the objects were not on the same plane did not effect the measurements.

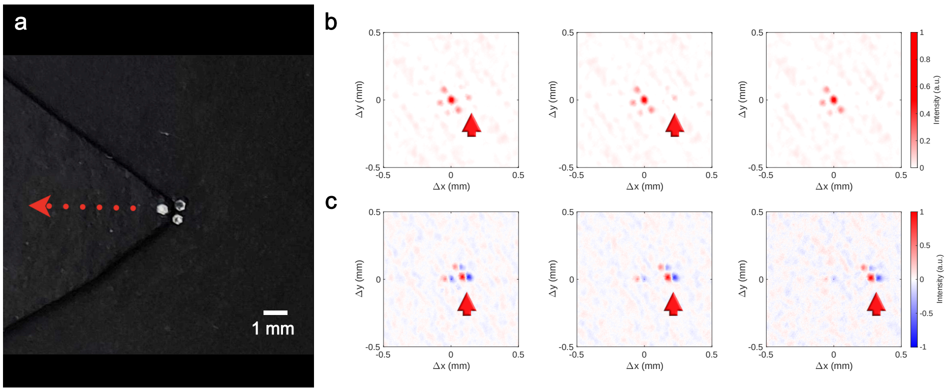

Figure S2: Experimental results of tracking moving reflective objects through scattering media via speckle correlations. **a** The object was made by two superimposed layers of substrate and three ~0.5 mm diameter flat reflective hexagons of biodegradable glitter. The top layer is movable and contains one hexagon, while the bottom layer is fixed and contains the other two hexagons. **b** Cross-correlation of the measured speckle pattern $I(t_n)$ at different times. **c** Plot of $I(t_n) \star I(t_{n-1}) - I(t_n) \star I(t_n)$ (see Supplementary Video 6).

It is important to notice that equation S6 is derived under the assumption that the objects to be tracked emit (reflect) light more or less isotropically, while in our experiments we deal with an almost specular reflection, which is only partially mitigated by a diverging illumination. As a consequence of the experiment geometry, all objects need to be small, or the light coming from them will never reach the camera. Notice that this is just an artefact of the experimental geometry, and for diffusive reflectors, large scattering layers, and cameras with a wide angle of acceptance, the only limit to the size of the object to be tracked is given by the signal to noise ratio.

S3.1 Longitudinal movement

This method is designed to track transverse motion, but since it has an infinite depth of field, all objects will look equally in focus independently from how far they are (although further away objects are likely to appear dimmer). As a consequence this method does not have any "sectioning" capabilities like confocal microscopy do. That said, closer objects will occupy a wider angular range, and will thus appear bigger, while objects further away will occupy a smaller angular range, thus appearing smaller, much like what we are used to with our own vision [5]. Supplementary Video 7 shows a simulation of what we expect to see when the object is not simply translating, but also rotating or changing distance.

References

[1] Labeyrie, A. Attainment of diffraction limited resolution in large telescopes by Fourier analysing speckle patterns in star images. *Astron. & Astrophys.* **6**, 85–87 (1970).

[2] Akkermans, E. & Montambaux, G. *Mesoscopic physics of electrons and photons* (Cambridge University Press, 2007).

[3] Bertolotti, J., Van Putten, E., Blum, C. et al. Non-invasive imaging through opaque scattering layers. *Nature* **491**, 232–234 (2012).

[4] Katz, O., Heidmann, P., Fink, M. et al. Non-invasive single-shot imaging through scattering layers and around corners via speckle correlations. *Nature Photon* **8**, 784–790 (2014).

[5] Hofer, M., Soeller, C., Brasselet, S. et al. Wide field fluorescence epi-microscopy behind a scattering medium enabled by speckle correlations. *Opt. Express* **26**, 9866–9881 (2018).
