# Supplementary material for: Tracking moving objects through scattering media via speckle correlations
Source: Nat Commun. 2022 Oct 1;13:5779. doi: 10.1038/s41467-022-33470-y (PMC9526741; doi:10.1038/s41467-022-33470-y)
Supplement: Supplementary file 3 — Description of Additional Supplementary Files [file 41467_2022_33470_MOESM3_ESM.pdf]

Supplementary Video 1: Experimental results of tracking moving objects via speckle correlations in the case of few objects in the scene. **a** Displayed intensity patterns on the DMD. **b** The autocorrelation of the speckle pattern measured at different times, and **c** the cross-correlation between the measured speckle at time t_0 and that at time t_n.

Supplementary Video 2: Experimental results of tracking moving objects via speckle correlations in the case of a lot of objects in the scene. **a** Moving scene makes up of many static dots and a single moving star on DMD. **b** The cross-correlation between the measured speckle patterns at t_0 and the speckle patterns at successive times. **c** Plot of $I(t_n) \star I(t_{n-2}) - I(t_n) \star I(t_n)$, where $I(t_n)$ is the measured speckle pattern at frame n. The dashed circle in the last two panels shows the memory range.

Supplementary Video 3: **a** Moving scene composed of many static dots and a single moving star. **b** Plot of $I(t_n) \star I(t_{n-3}) - I(t_n) \star I(t_n)$, **c** $I(t_n) \star I(t_{n-2}) - I(t_n) \star I(t_n)$ and **d** $I(t_n) \star I(t_{n-1}) - I(t_n) \star I(t_n)$, where $I(t_n)$ is the measured speckle pattern at frame n.

Supplementary Video 4: Numerical simulation of tracking moving objects via speckle correlations. **a** Moving object on a static background. **b** The convolution product between the moving scene in **a** and a random speckle pattern. **c** The autocorrelation of the moving scene in **a**. **d** Plot of $O(t_n) \star O(t_{n-1}) - O(t_n) \star O(t_n)$.

Supplementary Video 5: Experimental results of tracking moving objects via speckle correlations. **a** Displayed intensity patterns on the DMD. **b** The cross-correlation between the measured speckle patterns at t_0 and the speckle patterns at successive times. **c** Plot of $I(t_n) \star I(t_{n-1}) - I(t_n) \star I(t_n)$, where $I(t_n)$ is the measured speckle pattern at frame n.

Supplementary Video 6: Experimental results of tracking moving reflective objects through scattering media. **a** Measured speckle patterns $I(t_n)$. **b** The cross-correlation between the measured intensity at t_0 and the subsequent at $t_{n.}$ **c** Plot of $I(t_n) \star I(t_{n-1}) - I(t_n) \star I(t_n)$, where $I(t_n)$ is the measured speckle pattern at frame n.

Supplementary Video 7: Numerical simulation of **a** moving arrow over static points. In this case, the movement to follow includes lateral translations, rotations, and changes in size. **b** Plot of $I(t_n) \star I(t_{n-1}) - I(t_n) \star I(t_n)$, where $I(t_n)$ is the measured speckle pattern at frame n.
